# Case report: Urothelial injury in a female with breast cancer: a rare adverse event after the combination of paclitaxel and trastuzumab

**DOI:** 10.3389/fonc.2023.1258474

**Published:** 2024-01-18

**Authors:** Tinghua Cao, Zhipei Duan, Jing Zhu, Jing Wu, Jun Chen, Mingjun Jiang, Xialiang Lu, Yan Li

**Affiliations:** ^1^Department of Oncology, Suzhou Ninth People’s Hospital, Suzhou Ninth Hospital Affiliated to Soochow University, Suzhou, China; ^2^Department of Urinary Surgery, Suzhou Ninth People’s Hospital, Suzhou Ninth Hospital Affiliated to Soochow University, Suzhou, China; ^3^Department of Pathology, Suzhou Ninth People’s Hospital, Suzhou Ninth Hospital Affiliated to Soochow University, Suzhou, China

**Keywords:** trastuzumab, paclitaxel, urothelial injury, adverse events, urinary tract infection, case report

## Abstract

Several breast cancer (BC) patients showed urinary tract infection after adjuvant trastuzumab plus paclitaxel, but no case of urothelial injury has been reported. In this case, we report a 47-year-old female patient with stage I invasive ductal carcinoma in the left breast presenting with urothelial injury after the combination of trastuzumab and paclitaxel. Initially, the patient was highly suspected of having urinary tract infection as she showed abdominal and low back pain, as well as urinary irritation symptoms and hematuria. Unfortunately, the conditions were not attenuated after anti-infection therapy. Contrast-enhanced CT showed extensive exudation and edema in the bilateral renal pelvis, ureter, and bladder, together with dilatation and effusion in the renal pelvis and ureter. Cystoscopy showed extensive congestion, edema, and erosion in the bladder epithelium. Pathological analysis demonstrated slight thinning or even loss in the uroepithelial cell layer and interstitial congestion. In addition, there was growth arrest in the epithelial cells. Immunohistochemistry indicated HER2 expression in the urothelial cells. Finally, the patient was diagnosed with urothelial injury after combination of paclitaxel and trastuzumab. The symptoms were spontaneously cured with no administration of any antibiotics in the 3-month follow-up.

## Introduction

The adjuvant paclitaxel and trastuzumab has been commonly utilized for treating breast cancer (BC), which has greatly reduced the risk of recurrence and improved the patient’s survival (1, 2). Inevitably, many patients experience mild or even severe adverse events (AEs). Several BC patients show symptoms of urinary tract infection after trastuzumab and paclitaxel (3), but few or even no cases showed urothelial injury sharing similar symptoms with urinary tract infection (4). We hypothesized that the possibility that urothelial injury was misdiagnosed as urinary tract infection may help explain this. In this study, we reported a case of a 47-year-old female BC patient presenting with urothelial injury after paclitaxel and trastuzumab combination, which was initially misdiagnosed as urinary tract infection. This case report may contribute to our understanding of the AEs involving the urinary tract.

## Case presentation

**Figure 1:**
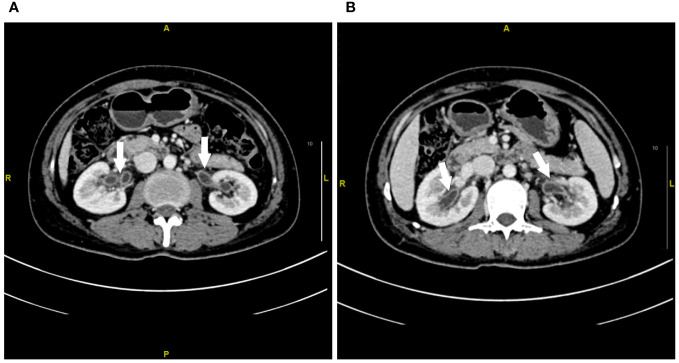
Contrast-enhanced CT imaging of the renal pelvis at week 1 after urinary tract irritation. **(A, B)** Extensive exudation and edema in the bilateral renal pelvis. The white arrow represents the edema of the renal pelvis.

**Figure 2:**
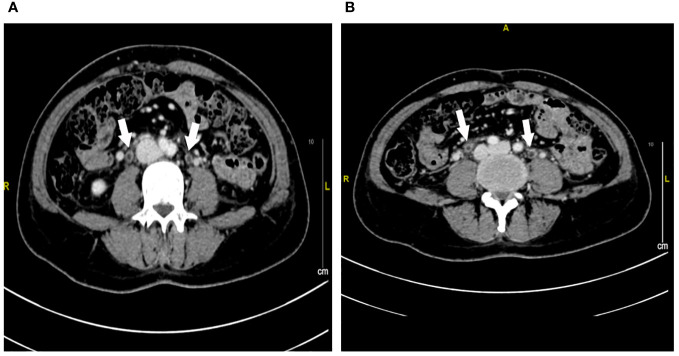
Contrast-enhanced CT imaging of the ureter at week 1 after urinary tract irritation. **(A, B)** Extensive exudation and edema in the bilateral ureter. The white arrow represents the edema of the ureter.

**Figure 3:**
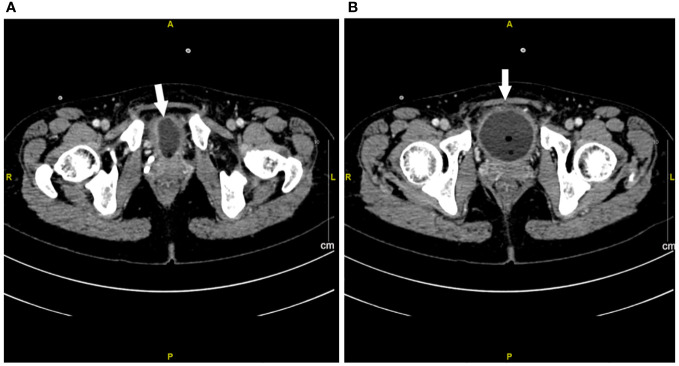
Contrast-enhanced CT imaging of bladder at week 1 after urinary tract irritation. **(A, B)** Extensive exudation and edema in bladder. The white arrow represents the edema of the bladder.

**Figure 4:**
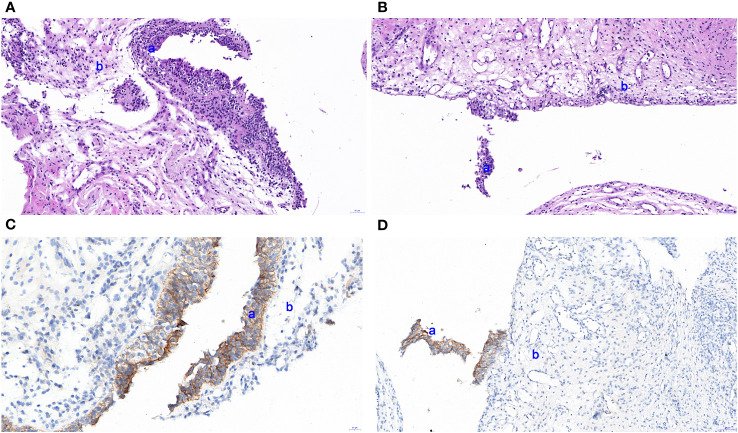
HE staining based on a urothelial biopsy specimen and IHC for HER2 expression in the urinary tract. **(A, B)** Slight thinning or even loss in the epithelial cell layer of the urinary tract. **(C, D)** IHC indicated slight thinning or loss of epithelial cells detected by pathological EnVision method staining. a and b represent the transitional cell and inherent layer of connective tissue, respectively. The images were observed under a magnification of ×100.

A 47-year-old lady with BC presented to our hospital for treatment. Before presenting to our department, she received modified radical resection in a hospital in Shanghai. Postoperative pathological results showed invasive ductal carcinoma (stage IA, pT1cN0M0), with no lymph node involvement. Immunohistochemistry (IHC) test results were estrogen receptor (ER) negative and progesterone receptor (PR) negative but HER2 positive. After excluding the contraindications of chemotherapy, the patient was given adjuvant therapy using paclitaxel and trastuzumab (5). On day 3, the patient showed abdominal and low back pain, as well as urinary irritation symptoms (e.g., frequent and urgent urination, dysuria) and hematuria. Routine blood test indicated neutropenia. On this basis, the patient was highly suspected of having urinary tract infection. She received ceftazidime and levofloxacin for 3 days. As the conditions showed no attenuation, she was switched to Tylenol anti-infective therapy. The results for urine bacterial and fungal cultures and smear microscopy in tuberculosis were negative. Urinary ultrasound showed hydronephrosis. Unfortunately, the conditions were not attenuated after 1-week anti-infection therapy. Antibiotics were then terminated and replaced with dexamethasone. Cystoscopy was completed, and a biopsy was done 6 days later with pathologic testing. Contrast-enhanced CT indicated extensive exudation and edema in the bilateral renal pelvis, ureter, and bladder, together with dilatation and effusion in the renal pelvis and ureter (**Figures 1**–**3**). Cystoscopy showed extensive congestion, edema, and erosion in the bladder epithelium. Urinary tract pathology indicated slight thinning or even loss in the epithelial cell layer of the urinary tract, together with interstitial congestion. In addition, there was growth arrest in the epithelial cells, with a high possibility of cellular exfoliation. IHC indicated HER2 positivity in the urinary tract (**Figure 4**). Finally, the patient was diagnosed with urothelial injury rather than urinary tract infection after combination of paclitaxel and trastuzumab. In the 3-month follow-up, the symptoms were spontaneously cured with no administration of any antibiotics. This study was performed according to the convention of the Declaration of Helsinki. The research protocol was approved by the Ethics Committee of Suzhou Ninth People’s Hospital, Suzhou Ninth Hospital Affiliated to Soochow University. Written informed consent was obtained from the patient.

## Discussion

Urinary tract infection has been commonly reported in patients who underwent combined trastuzumab and paclitaxel. Nevertheless, no studies have reported cases with urothelial injury after such a regimen. In this study, our patient was initially misdiagnosed with urinary tract infection as she showed symptoms of urinary irritation. For a typical lower urinary tract infection, a 3-day anti-infection therapy regime is generally sufficient. Conversely, complex cases involving the upper urinary tract often require a prolonged antibiotic course, usually spanning 7 days to 14 days. The efficacy of treatments for urinary tract infections is commonly discernible within an approximate timeframe of 3 days. In cases of poor response to 3- or 7-day antibiotic therapy, subsequent evaluations (including repeated routine urinalyses, midstream urine cultures, and enhanced CT scans when necessary) successfully excluded the possibility of urinary tract infection. Bladder biopsy, together with the spontaneous cure, confirmed the possibility of urothelial injury induced by trastuzumab or paclitaxel. This case report may enhance our understanding of the AEs involving the urinary tract after trastuzumab or paclitaxel.

To investigate whether the urothelial injury was induced by trastuzumab and/or paclitaxel, we investigated the metabolism of these two drugs based on the previous literature. The total prototype drug in urine after paclitaxel administration was 1.5%–9% of the administered dose; the majority was eliminated by the organs except kidney (6), with a half-life of merely 6–13 h (7). Therefore, complete elimination was achieved after a period that was about 5.0-fold of the half-life. In contrast, the half-life for trastuzumab was 28–38 days, with a subsequent clearance period of up to 27 weeks (8). Meanwhile, the urothelial injury was completely cured at month 4, which could be explained by the pharmacokinetics of trastuzumab rather than paclitaxel. Moreover, the patient showed expression of HER2 on the uroepithelial cells, together with decreased proliferation, thinning, or even loss of bladder epithelial cells. These validated that the urothelial injury was induced by trastuzumab.

The exact mechanism of trastuzumab-induced AEs in urinary tract is still unclear due to the rarity of studies. HER2 expression has been frequently detected in the organs or tissues involved by trastuzumab-induced AEs. According to The Human Protein Atlas website on HER2 expression in partial normal tissues (9), *HER2* RNA and protein were detected in human skin, pulmonary tissues, digestive tract, and cardiac tissues, respectively. Indeed, trastuzumab has been well acknowledged to induce AEs in these sites. HER2-targeted therapies (e.g., trastuzumab, lapatinib, pertuzumab) have been reported to carry risks of cardiopulmonary, hematologic, gastrointestinal, and other AEs (2). HER2 plays crucial roles in several biological processes in normal tissues, such as cellular differentiation, necrosis, and proliferation (10). Trastuzumab-induced AEs may be associated with its interaction with HER2 in these cells. For instance, trastuzumab-induced cardiotoxicities may be associated with the interaction between trastuzumab and HER2 expressed in cardiomyocytes that is necessary for the maintenance of cardiac structure and function (11). In addition, as HER2 is involved in the maintenance of membrane integrity in normal gastrointestinal tissues; the inhibition of HER2 expression induced by trastuzumab may lead to a range of gastrointestinal AEs in HER2-overexpressing BC patients (10). HER2 is also expressed in normal urothelial cells. In this case, the patient showed high HER2 expression, and trastuzumab may interact with HER2 expressed in normal cells, resulting in the occurrence of urothelial injury.

Trastuzumab has been well acknowledged as an anti-HER2 drug, and there are ample evidence-based and real-world data on safety. Individual differences and co-administration often lead to unrecognized AEs, which can affect the correct diagnosis and treatment adjustment. The case reported herein suggests that trastuzumab can directly cause urothelial injury, the severity of which may be related to the level of HER2 expression within the individual and the dose of the drug used. This AE has not been reported before, and this case report can provide a basis for clinical recognition and management of this AE in a timely manner. Additionally, this helps promote further research on the mechanisms related to the AEs of targeted therapeutic agents, which can better contribute to the safety of clinical treatment.

## Conclusion

In this case report, we report a rare case showing urothelial injury after combined therapy based on trastuzumab and paclitaxel. It was induced by trastuzumab based on the drug metabolism analysis, together with the expression of HER2 on the urinary epithelial cells, decreased proliferation, thinning, or even loss of bladder epithelial cells. Meanwhile, we hope to raise attention on differential diagnosis between urothelial injury and infection after the combination of trastuzumab and paclitaxel.

## Data availability statement

The original contributions presented in the study are included in the article/supplementary material. Further inquiries can be directed to the corresponding author.

## Ethics statement

The studies involving humans were approved by Suzhou Ninth People’s Hospital, Suzhou Ninth Hospital Affiliated to Soochow University. The studies were conducted in accordance with the local legislation and institutional requirements. The participants provided their written informed consent to participate in this study. Written informed consent was obtained from the individual(s) for the publication of any potentially identifiable images or data included in this article.

## Author contributions

TC: Formal analysis, Visualization, Writing – original draft. ZD: Formal analysis, Writing – review & editing. JZ: Formal analysis, Writing – review & editing. JW: Formal analysis, Writing – review & editing. JC: Investigation, Writing – review & editing. MJ: Investigation, Writing – review & editing. XL: Investigation, Writing – review & editing. YL: Conceptualization, Writing – review & editing.
